# A school based study of time trends in food habits and their relation to socio-economic status among Norwegian adolescents, 2001–2009

**DOI:** 10.1186/s12966-014-0115-y

**Published:** 2014-09-25

**Authors:** Anne-Siri Fismen, Otto Robert Frans Smith, Torbjørn Torsheim, Oddrun Samdal

**Affiliations:** Department of Health Promotion and Development, University of Bergen, Christiesgate 13, Bergen, 5015 Norway; Division of Mental Health, Norwegian Institute of Public Health, Kalfarveien 31, Bergen, 5018 Norway; Department of Psychosocial Science, University of Bergen, Christiesgate 12, Bergen, 5015 Norway

**Keywords:** Food habits, Fruit, Vegetables, Sweets, Soft drink, Adolescence, Socio-economic inequality, FAS

## Abstract

**Background:**

In recent years, adolescents’ food habits have become a major source of concern, and substantial policy and intervention efforts have been made to influence adolescents to consume more fruit and vegetables and less sweets and soft drink. Particular attention has been devoted to the social gradient in food habits, aiming to reduce dietary inequality. However, few internationally published studies have evaluated trends in teenagers’ food habits, or investigated how dietary inequalities develop.

**Methods:**

We used Norwegian cross-sectional data from the international Health Behaviour in School-Aged Children (HBSC) study, collected via three nationally representative and comparable questionnaire surveys in 2001, 2005 and 2009. Food habits were identified by students’ consumption of fruit, vegetables, sweets and sugar rich soft drink. Socio-economic status (SES) was measured with the Family Affluence Scale (FAS). Multilevel logistic regression was used to analyze the data.

**Results:**

The analyses indicated an overall positive trend in food habits among adolescents in Norway. Students were more likely to consume fruit (OR 1.76, CI 1.61-1.92) and vegetables (OR 1.51, CI 1.37-1.66) daily in 2005 as compared to 2001, and were less likely to consume sweets (OR 0.58, CI 0.51-0.66 resp. OR 0.77, CI 0.67-0.90) and soft drink (OR 0.55, CI 0.49-0.62 resp. OR 0.84, CI 0.73-0.96) daily when comparing, respectively, 2005 with 2001 and 2009 with 2005. Across all survey years, students with higher SES were more likely to eat fruit (OR 1.47, CI 1.32-1.65) and vegetables (OR 1.40, CI 1.24-1.58) daily than did students with lower SES. Our analyses indicated that the socio-economic differences were stable in the period 2002 - 2010, with uniform improvement in fruit and vegetable consumption across all SES levels. No significant associations between SES and intake of sweets and sugar-added soft drink were found.

**Conclusion:**

The study identifies an overall improvement in diet among adolescents over a period characterized by onset of as well as ongoing initiatives targeting young people’s food habits. However, the observed socio-economic gradient in fruit and vegetable consumption remained unchanged.

## Background

Adolescents’ food choices and diets have become a major source of concern in recent decades. This must be viewed in the context of western countries’ high prevalence of overweight [1–3] and chronic diseases [4–6], and the link to diet [7–10]. Although chronic diseases are not very prevalent among teenagers, there is emerging evidence that there is early onset of risk factors, even in adolescence [11]. Improved food habits among this age group are likely to reduce the population-based incidence of chronic diseases, because food habits established at an early age extend into adulthood [12–14]. Food habits are also highly relevant in terms of social inequalities in health, because dietary inequality is linked to health inequality [15]. This is of particular concern in light of increased recognition that tackling health inequality is a key public health challenge in the twenty-first century.

Frequent consumption of fruit and vegetables accompanied by a low intake of simple sugar (<10% of total energy) is widely recommended by international nutritional authorities [16–18]. However, many schoolchildren fall short of these recommendations [19–24]. Poor diet is of particular concern with regard to adolescents living in families of low socio-economic status (SES), because adolescents of low SES eat less fruit and vegetables [20,25–28] and more sugar-added food items [24,29,30] than their high SES counterparts. Based on this background, initiatives to improve and equalize young people’s food habits receive high priority in most Western countries [16,17].

In Norway, improving adolescents’ food habits is a national policy goal [31]. Substantial efforts have been made to change the consumption patterns of Norwegian teenagers, who are among the lowest fruit and vegetable consumers in Europe [32] and high consumers of sweetened beverages [33]. Various initiatives, such as information campaigns, school text pamphlets, television and radio announcements and advertisements, have been employed by the national Nutritional Council, often in close collaboration with other organizations and agencies. In order to reach students across all SES groups, several national and regional educational interventions targeting students’ food habits have been trialed in Norwegian schools [34–37]. There has been a systematic prioritization of providing fruit and vegetables at schools [38] and in sport halls, and sugar-free mineral water has replaced sugar-added soft drink in vending machines in the vast majority of public schools [34]. Also the grocery industry has been proactive in its marketing of fruit and vegetables, and some grocers reduced the prices of fruit and vegetable by waiving the taxes on fruit and vegetables in 2007, rather than passing them to the consumers. In 2007, a nationwide free (not parent-funded) school fruit program was implemented in all Norwegian secondary schools (grades 8–10) and all combined schools (grades 1–10) in 2007. This is possibly the most comprehensive and costly (230 million NKR/27 million Euro in 2011) initiative targeting Norwegian adolescents’ consumption of fruit and vegetables, and it provides all children in lower secondary schools and combined schools with a free piece of fruit or a carrot every day. Marketing of sweets and beverages to young people is strictly prohibited in Norway [39,40] and the government has moreover imposed the world’s highest economic fees on soft drink and other non-alcoholic beverages.

Despite all the attention devoted to adolescents’ food habits in both Norway and other European countries [41,42], few internationally published studies have evaluated nutrition policies and educational campaigns aimed at teenagers. Moreover, few studies have reported trends in young people’s consumption of fruit, vegetables and sugar-rich products such as sweets and soft drinks. The number of studies that report on the development of dietary inequalities is even more limited. Studies of trends in adolescents’ food habits are highly relevant for the evaluation of nutrition policies and dietary interventions, as well as for assessing the need for further initiatives aimed at improving and equalizing eating habits. Such studies are of international interest because they provide knowledge about how policies and interventions influence teenagers’ food habits. Studies of food habits should moreover measure different items individually. Combining fruit and vegetable intake as a single measurement, for example, does not facilitate assessments of whether consumption is meeting the recommendations for fruit and for vegetables individually. As the determinants of fruit intake and vegetable intake may differ, interventions may be more beneficial if tailored specifically for fruit and for vegetable consumption, separately. Moreover, the effects of SES have been shown to vary among food items [25,28,43]. Different food items should therefore be studied individually, in terms of both intake recommendations and socio-economic inequality. Differentiation between specific foods is needed to examine individual trends separately and to follow the social gradient more accurately.

In the present study, we evaluated how trends in the consumption of fruit, vegetables, sweets and soft drink developed among Norwegian adolescents in the period 2001–2009, and how dietary inequalities developed during this period. Building on the systematic strategies and initiatives aimed at improving the food habits and reducing dietary inequalities, improved dietary patterns across all SES groups has been greatly anticipated. We used cross-sectional data from the Health Behavior in School-Aged Children (HBSC) study collected via three nationally representative and comparable questionnaire surveys in 2001, 2005 and 2009. The study population consisted of Norwegian schoolchildren aged 11, 13, 15 and 16 years. As far as we know, nationally representative Norwegian data on such trends have never been published internationally.

## Methods

We used data from the Norwegian contribution to the WHO cross-national HBSC study (www.hbsc.org) collected in 2001/2002, 2005/2006 and 2009/2010. A stratified standard cluster sampling procedure was used to ensure national representativeness [44] when selecting the data. School class (classroom unit) was the primary sampling unit, with one participating class per age group in each selected school. A standard cluster sampling procedure based on a geographically stratified list with sequential selection from a randomized starting point was used to select the sample. The overall school, school classes and student response rate was respectively in 73%, 88% and 89% in survey year 2001, 69%, 69% and 84% in survey year 2005 and 55%, 56% and 81% in survey year 2009. Non-responses comprised primarily either class- or school-level, non-participation or student absence on the day the survey was administered. Participation was based on passive parental consent. The students answered the questionnaire in the classroom after receiving standardized instructions from their teacher [44]. Participation was anonymous. The students were asked not to write their names on the questionnaire and to return the completed questionnaire in the provided envelope after sealing it. The Privacy Ombudsman at the Norwegian Social Science Services gave assurance that the study complied with Norwegian privacy and confidentiality requirements. The Norwegian Western Regional Ethical Committee determined that the study did not require ethical clearance.

### Questionnaire

The questionnaire was developed through international consensus and translated into the languages of the participating countries. Back-translation to English was approved by independent researchers in the international study [44].

### Socio-economic status

#### The Family Affluence Scale (FAS)

The FAS is a measure of material affluence derived from the characteristics of the family’s household. It consists of the following four items: **1.** “Does your family own a car, van or truck?” (No = 0; One = 1; Two or more = 2). This item is a component of the Scottish Deprivation Index developed by Carstairs and Morris [45], which is used widely in health inequalities research. **2.** “How many times have you travelled away on holiday with your family during the past 12 months?” (Never = 0; Once = 1; Twice = 2; Three or more times = 3). This item is a measure of “deprivation of home facilities” [46]. **3.** “Do you have a bedroom to yourself?” (No = 0; Yes = 1). This item is a proxy for overcrowding, classified by Townsend [46] as housing deprivation, and is also a component of the Scottish Deprivation Index. **4.** “How many computers does your family own?” (None = 0; One = 1; Two = 2; More than two = 3). This item was introduced into the FAS to better differentiate between SES groups in affluent countries [47]. These FAS items provide a scale that can be easily completed by students [48].

#### Ridit transformation of FAS

A central assumption of the present study is that the FAS has at least ordinal measurement properties, and that the scores can be used to rank individuals and groups along a latent continuum of material wealth. When assuming that the ordered categorical variable approximates an underlying, but not measurable, continuous variable, ridit transformation analysis may be used [49]. Ridit transformation converts ordered categorical responses to cumulative probabilities and is a widely used approach for SES scales with ordinal measurement [50,51]. It has been used in previous HBSC studies [25,52]. In the present study, the FAS was transformed to yield a continuous material deprivation score ranging from 0 to 1, with a whole-sample mean of 0.5. The material deprivation score reflects a student’s level of family affluence relative to the rest of the cohort. A student with a deprivation score of 1 is at the top of the material hierarchy (100% of the other students have a lower level of family affluence), whereas a student with a score of 0 is at the bottom of the material hierarchy (no other student has a lower level of family affluence). In the predictive model, with different eating habits as dependent variables, the regression coefficient of the FAS score can be directly interpreted as the predicted difference in eating habits between the least deprived individual and the most deprived individual. This valuable property has been exploited in a series of studies using ordinal SES ratings [50,51,53].

### Eating habits

The levels of consumption of fruit, vegetables, sweets and sweetened soft drink were measured by items with the same wording: “How many times a week do you consume fruit/vegetables/sweets/ sweetened soft drink?” (Never = 0; Less than once a week = 1; Once a week = 2; Two to four times a week = 3; Five to six times a week = 4; Once a day = 5; More than once a day = 6). Students who ate fruit, vegetables, sweets or sugar-added soft drink at least once daily were identified by the responses: “Once a day” and “More than once a day”. These responses (categories 4 and 5) were referred to as daily consumption and recoded as 1. Responses to categories 0–3 were recoded as 0.

The aim of the HBSC study is to evaluate health behavior and lifestyle patterns. The outcome variables were dichotomized to measure whether or not the students included fruit and vegetables, as well as sweets and soft drinks, in their everyday diet. The variables have been treated similarly also in other studies [25,28,54] within the HBSC network.

### Statistical analysis

We calculated standardized frequencies of daily consumption of fruit, vegetables, sweets and soft drink for each gender and age group for each year of data collection. Backward difference coding was used to examine the effects of survey year (2005 versus 2001, and 2009 versus 2005). The four measures (daily fruit/vegetables/sweets/soft drink consumption) were further analyzed separately by multilevel logistic regression models. School class was included as a random effect. Intraclass correlations were derived from the unconditional models and equaled 0.07, 0.05, 0.10 and 0.13 for respectively daily fruit, vegetables, sweets and soft drink consumption. The results for each outcome were estimated by two models. In model 1, we analyzed the main effects of age, gender, SES and survey year. In model 2, interactions between survey years and gender, age and SES were tested. Estimation of trends was based on analyses of changes in the rates of daily consumption of the four food items calculated for each year. Odds Ratios (ORs) with 95% confidence intervals (CIs) indicated the likelihood of daily consumption of fruit, vegetables, sweets and soft drink, with certain characteristics relative to the reference group. The reference group for gender was boys. The reference group for age was 11-year-olds.

All statistical analyses were performed in IBM SPSS version 20.

## Results

### Time trends in food habits

Our analyses indicated an overall positive trend in food habits among adolescents in Norway. Compared with 2001, more students in 2009 reported daily consumption of fruit and vegetables and fewer reported daily consumption of sweets and soft drink.

As shown in Table 1, the improvements in food habits were greatest in the 2001 to 2005 period. Compared with 2001, the number of students reporting daily consumption of fruit and vegetables in 2005 increased by 45% and 35%, respectively. However, the analyses identified no further improvements in the 2005 to 2009 period. Students’ intake of sweets and soft drink was found to have decreased more consistently, with significant reductions between 2001 and 2005, as well as between 2005 and 2009. The number of students reporting daily consumption of sweets and soft drink decreased by 51% and 45%, respectively, during the 2001 to 2009 period. The results of the frequency analyses are supported by the multilevel logistic regression analyses presented in Tables 2 and 3, with significant changes in the rates of daily consumption of fruit (OR 1.76, CI 1.61-1.92, p < .001) and vegetables (OR 1.51, CI 1.37-1.66, p < .001) between 2001 and 2005. Significant changes in the consumption of sweets were found when comparing 2001 with 2005 (OR 0.58, CI 0.51-0.66, p < .001) and 2005 with 2009 (OR 0.78, CI 0.67-0.90, p = .001), and in soft drink consumption between 2001 and 2005 (OR 0.55, CI 0.49-0.62, p < .001) and between 2005 and 2009 (OR 0.84, CI 0.73-0.96, p = .012).

**Table 1:** **Gender- and age-standardized frequencies of food habits by survey year**

	**2001**	**2005**	**2009**	**Effect of year: 2005 vs 2001**			**Effect of year: 2009 vs 2005**		
	**% (n)**	**% (n)**	**% (n)**	**OR**	**95% CI**	**p-value**	**OR**	**95% CI**	**p-value**
Daily fruit	26.5 (1863)	38.5 (2477)	37.9 (2180)	1.76	1.61–1.92	<.001	0.97	0.87–1.06	.468
Daily vegetables	20.6 (1444)	28.1 (1808)	28.3 (1630)	1.51	1.37–1.66	<.001	1.01	0.92–1.11	.823
Daily sweets	16.2 (1138)	10.1 (649)	7.9 (457)	0.58	0.51–0.66	<.001	0.77	0.67–0.90	.001
Daily soft drink	22.2 (1561)	13.8 (889)	12.1 (697)	0.55	0.49–0.62	<.001	0.84	0.73–0.96	.001

OR – odds ratio; CI – confidence interval.

**Table 2:** **Generalized linear mixed model of daily consumption of fruit and vegetables with fixed effects**

	**Daily fruit**						**Daily vegetables**					
	**Model 1**			**Model 2**			**Model 1**			**Model 2**		
Fixed effects	OR	95% CI	p-value	OR	95% CI	*p*-value	OR	95% CI	*p*-value	OR	95% CI	*p*-value
Intercept	0.47	0.43- 0.51	<.001	0.48	0.44-0.53	<.001	0.32	0.29-0.35	<.001	0.32	0.29-0.36	<.001
Female	1.82	1.71-1.94	<.001	1.72	1.53-1.95	<.001	1.46	1.36-1.57	<.001	1.43	1.26-1.62	<.001
Age = 16	0.43	0.38-0.47	<.001	0.36	0.31-0.42	<.001	0.57	0.51-0.64	<.001	0.48	0.42-0.56	<.001
Age = 15	0.62	0.56-0.69	<.001	0.59	0.52-0.68	<.001	0.67	0.61-0.75	<.001	0.67	0.57-0.77	<.001
Age = 13	0.73	0.66-0.81	<.001	0.77	0.67-0.88	<.001	0.76	0.68-0.84	<.001	0.83	0.72-0.96	.011
2005 vs 2001	1.76	1.61-1.92	<.001	1.99	1.61-2.46	<.001	1.51	1.37-1.66	<.001	1.67	1.30-2.15	<.001
2009 vs 2005	0.97	0.89-1.06	.486	0.89	0.72-1.11	.312	1.01	0.92-1.11	.793	1.00	0.78-1.30	.977
SES	1.47	1.32-1.65	<.001	1.47	1.31-1.64	<.001	1.40	1.24-1.58	<.001	1.41	1.24-1.60	<.001
Female × age = 16				1.34	1.10-1.62	.003				1.34	1.12-1.62	.002
Female × age = 15				1.09	0.91-1.30	.346				1.02	0.84-1.24	.853
Female × age = 13				0.92	0.77-1.10	.336				0.84	0.70-1.01	.060
Female × 2005/2001				0.94	0.81-1.10	.422				0.97	0.82-1.15	.676
Female × 2009/2005				1.07	0.91-1.25	.402				0.92	0.78-1.08	.291
Age = 16 × 2005/2001				0.86	0.67-1.10	.238				0.92	0.70-1.21	.562
Age = 15 × 2005/2001				0.96	0.75-1.22	.746				1.04	0.80-1.36	.757
Age = 13 × 2005/2001				0.85	0.68-1.08	.183				0.93	0.72-1.22	.612
Age = 16 × 2009/2005				0.96	0.74-1.24	.737				1.13	0.85-1.48	.406
Age = 15 × 2009/2005				1.27	0.99-1.61	.054				1.19	0.92-1.53	.192
Age = 13 × 2009/2005				1.20	0.94-1.52	.141				1.00	0.78-1.29	.989
SES × 2005/2001				0.99	0.77-1.27	.920				0.90	0.68-1.18	.436
SES × 2009/2005			0.91	0.69-1.22	.538				0.98	0.71-1.36	.905	

OR – odds ratio; CI – confidence interval; the reference group for gender was boys and for age 11 year olds.

**Table 3:** **Generalized linear mixed model of daily consumption of sweets and soft drinks with fixed effects**

	**Daily sweets**						**Daily soft drink**					
	**Model 1**			**Model 2**			**Model 1**			**Model 2**		
Fixed effects	OR	95% CI	*p*-value	OR	95% CI	*p*-value	OR	95% CI	*p*-value	OR	95% CI	*p*-value
Intercept	0.07	0.06-0.09	<.001	0.07	0.06-0.09	<.001	0.13	0.11-0.14	<.001	0.12	0.10-0.14	<.001
Female	1.03	0.94-1.12	.564	1.15	0.92-1.44	.229	0.58	0.53-0.63	<.001	0.61	0.50-0.74	<.001
Age = 16	2.11	1.80-2.46	<.001	2.21	1.78-2.73	<.001	2.82	2.44-3.26	<.001	3.10	2.59-3.70	<.001
Age = 15	2.02	1.72-2.38	<.001	2.30	1.84-2.87	<.001	2.53	2.18-2.93	<.001	2.57	2.14-3.08	<.001
Age = 13	1.55	1.31-1.85	<.001	1.66	1.31-2.01	<.001	1.65	1.41-1.93	<.001	1.61	1.33-1.95	<.001
2005 vs 2001	0.58	0.51-0.66	<.001	0.38	0.26-0.54	<.001	0.55	0.49-0.62	<.001	0.52	0.38-0.72	<.001
2009 vs 2005	0.78	0.67-0.90	.001	1.36	0.87-2.16	.167	0.84	0.73-0.96	.012	0.92	0.63-1.34	.667
SES	0.98	0.84-1.16	.844	0.94	0.77-1.15	.561	0.93	0.81-1.07	.327	0.92	0.79-1.07	.268
Female × age = 16				1.01	0.77-1.32	.971				0.83	0.65-1.06	.137
Female × age = 15				0.81	0.61-1.07	.141				0.97	0.76-1.25	.826
Female × age = 13				0.85	0.64-1.12	.247				1.02	0.79-1.33	.854
Female × 2005/2001				1.27	1.03-1.56	.024				1.12	0.92-1.37	.253
Female × 2009/2005				0.82	0.63-1.01	.128				0.85	0.67-1.08	.181
Age = 16 × 2005/2001				1.67	1.15-2.43	.008				1.12	0.79-1.56	.526
Age = 15 × 2005/2001				1.56	1.08-2.31	.019				1.11	0.79-1.57	.540
Age = 13 × 2005/2001				1.22	0.82-1.84	.331				1.04	0.72-1.50	.848
Age = 16 × 2009/2005				0.70	0.46-1.09	.112				1.05	0.71-1.55	.797
Age = 15 × 2009/2005				0.59	0.38-0.94	.025				0.92	0.62-1.36	.676
Age = 13 × 2009/2005				0.56	0.34-0.92	.023				0.83	0.54-1.27	.394
SES × 2005/2001				0.95	0.66-1.36	.767				0.87	0.64-1.21	.419
SES × 2009/2005				0.84	0.49-1.44	.517				1.04	0.69-1.57	.843

OR – odds ratio; CI – confidence interval; the reference group for gender was boys and for age 11 year olds.

### Socio-economic inequalities

Analyses of the bivariate associations between SES and food habits indicated a positive association between SES and the consumption of fruit (OR 1.40, CI 1.26-1.56, p < .001) and vegetables (OR 1.34, CI 1.20–1.51; p < .001); students with higher FAS scores were more likely to eat fruit and vegetables on a daily basis. These associations remained significant after taking into account gender, age, and time (see Table 2). We found no significant association between FAS score and the consumption of sweets and soft drink in the study population. As shown in Tables 2 and 3, the interaction effects between SES and eating habits were not significant, suggesting that the impact of SES on eating habits was stable between 2001 and 2005 and between 2005 and 2009.

### Age and gender differences in eating habits

Age differences were found for the consumption of all four food types. As shown in Tables 3 and 4, older students reported consuming less fruit and vegetables and more sweets and soft drink than younger ones. Girls were more likely to report daily consumption of fruit (OR 1.82, CI1.71-1.94, p < .001) and vegetables (OR 1.46, CI 1.36-1.57, p < .001) and less likely to report daily consumption of soft drink (OR 0.58, CI 0.53-0.63, p < .001) than were boys. The observed gender differences increased with for 16 year olds as compared to 11 year olds with regard to fruit (OR 1.34, CI 1.10-1.62, p = .003) and vegetable (OR 1.34, CI 1.12-1.62, p = .002) consumption. The association between age, gender and food habits remained stable over the whole 2001–2009 period except for the consumption of sweets, for which gender differences increased from 2001 to 2005 (OR = 1.27. CI 1.03-1.56, p = .024) and age differences first increased from 2001 to 2005 and then decreased from 2005 to 2009.

## Discussion

The study identified overall improved food habits during a period in which government and grocery industry initiatives targeting young people’s food habits were initiated and ongoing. The analyses indicated that survey year predicted improvements differently for different food items. Although the greatest improvements in all four outcomes were found in the 2001–2005 period, the consumption of sweets and soft drink decreased steadily, with significant improvements being registered across the entire period. By contrast, no further improvement in fruit and vegetable consumption was found between 2005 and 2009. At all three time points, students from low SES families reported lower intakes of fruit and vegetables than did students from high SES families. The survey year predicted no significant changes in the association between SES and the reporting of daily consumption of fruit and vegetables, suggesting that the socio-economic gradient has remained unchanged since 2001. SES was not a significant determinant for the consumption of sweets and soft drink in any of the years of data collection.

Increased fruit and vegetable consumption has previously been found in regional Norwegian samples in the period 2001–2008 [26,55], as well as in Scotland (2002–2006) [21] and Lithuania (2001–2009) [54], while increased fruit consumption has been found in Denmark (2001–2005) [56] and the Netherlands (2001–2009) [20]. Similarly, reduced intake of sugar-added soft drink has previously been found in a regional Norwegian sample (2001–2008) [29], in Scotland (2002–2006) [21] and the Netherlands (2001–2009) [20]. By contrast, increased consumption of soft drink was found in Lithuania (2001–2009) [54], Ireland (1997–2005) [57] and the USA (1999–2004) [58]. Our findings of age and gender differences are supported by other studies with regard to fruit and vegetables [21,28,54,56] and soft drink [28,29]. The finding that students in high SES groups are more likely to report daily consumption of fruit and vegetables is also supported by other studies [26,28,29]. Stable socio-economic inequalities in adolescents’ fruit and vegetable consumption have also been found in Scotland [21].

Our analysis documents increased fruit and vegetable consumption, and decreased intake of soft drinks and sweets, in the same period as a range of national initiatives aiming to improve food habits was established or maintained. The present study does not evaluate any specific structural incentives or educational interventions and cannot conclude whether the current nutrition policy has had an effect on adolescents’ food habits. However, initiatives similar to those characterizing the Norwegian nutrition policy are in the literature evaluated to influence food habits positively. For example, information campaigns like the Norwegian “5 a day” program, as well as nutrition education programs similar to those implemented in the Norwegian school, are found to improve food habits [59].

Our findings of improved food habits are encouraging. However, our analysis documented no further improvement in fruit and vegetable consumption when comparing survey year 2009 to 2005. Given the onset of a nationwide free school fruit program in 2007, and the fact that all students age 13 and 15 in this study cohort was provided with a piece fruit/carrot at school every day, this finding might be surprising. However, this finding indicates that although availability is an important determinant to fruit and vegetable consumption, availability alone is not enough to increase intake of fruit and vegetables. It should also be noted that time trends in fruit and vegetables consumption identified in this study cohort is quite similar to the development in fruit and vegetable intake in Norwegian population as a whole, where fruit consumption increased only slightly between 2003 and 2012, and vegetable consumption increased more steadily [60]. This consumption pattern may have influenced the availability of fruit in the home milieu and may contribute to explain why we found no further increased fruit intake when comparing 2009 to 2005. The present study does further document an extensive reduction in soft drink consumption. This does probably not reflect a reduction in the total intake of soft drink but rather a replacement of sweetened soft drink by sugar-free alternatives [29] as well as increased use of water on bottles [61].

According to our analyses, socio-economic inequalities in fruit and vegetable consumption remained unchanged in the period were several incentives aiming to reduce dietary inequalities were onset or ongoing. The observed socio-economic gradient might reflect the high cost of fruit and vegetables in Norway. It is shown that purchase of fruit and vegetables is price sensitive [62,63] and that high cost is considered as a barrier to increase the intake in low SES families [64]. Fruit and vegetables are indeed shown to be particularly strongly and consistently associated to socio-economic position in the Northern part of Europe, also over time [65]. With the high cost it might therefore be particularly challenging for lower SES families to respond to the Norwegian authorities’ recommendation of providing the whole family with “five a day”. However, reducing socioeconomic differences is a national goal in Norway and the politicians held particularly great expectations to the free school fruit program. Our results might therefore be disappointing. On the other hand, the uniform improvements in food habits across all SES groups is an encouraging outcome, as improvements in general population health are typically accompanied by increasing inequalities [66,67], with affluent groups achieving health improvements more easily than others. Our findings of no such widening of the inequality gap might indicate that socio-economic circumstances influence adolescents differently to adults, a perspective supported by Hanson and Chen [68], who found dietary inequality to be less robust during adolescence than adulthood.

Intake of sweets and soft drinks was not associated to SES in this study population. This is in line with other studies on adolescents’ food habits in western countries [25,28] and moreover in strikingly in contrast to studies on adolescents’ soft drink consumption in several Central and Eastern European countries [28,54]. This might, as pointed to by Vereecken [28], indicate that in Central and Eastern European countries, soft drinks are still considered luxury items, affordable only by higher SES groups. However, studies of socio-economic disparities should always be read in the context of the SES indicator that is used. This means that the association between SES and food habits among Norwegian adolescents might be different if the FAS were to be replaced with an indicator that taps other dimensions of the SES construct than material wealth. Soft drink consumption, for example, is associated with SES among Norwegian adolescents in studies that use parental education [29,30,69], parental occupation [28] or cultural capital [25] as indicators of SES. It is likely that soft drinks are less influenced by material wealth as it is relatively cheap compared to other food items in Norway. This emphasizes the complexity of conducting research to identify socio-economic inequality in eating behaviors, in particular among children and adolescents.

### Limitations

The study has limitations that should be considered when interpreting the findings. The use of the FAS could be criticized both methodologically and conceptually. The FAS was developed not to measure material capital among Norwegian adolescents in particular but to investigate the socio-economic circumstances of young people in all of Europe and North America. Computers are currently used in Norwegian school settings and for daily homework. Most teenagers therefore have their own computer. Low automobile ownership might reflect the environmental consciousness of parents rather than low SES. However, the FAS has been shown to be a valid instrument for measuring material circumstances among young people [70,71] and is currently used for this purpose [72]. Moreover, the use of ridit transformation compensates for some of the limitations, making the FAS a relevant SES indicator for Norwegian conditions.

Finally, the HBSC questionnaire has been recognized as a valid instrument in epidemiological studies ranking adolescents according to their usual food intake [73], and is therefore considered valid for measuring the intake of fruit, vegetables, sweets and soft drink. However, it should be noted that the questionnaire only assesses frequency of consumption. When assessing changes over time, changing portion sizes could potentially have influenced our results of dietary habits. Our study does not measure the amount of fruit, vegetable, sweets and soft drinks consumed by the students. The study identifies students’ report of frequencies of consumption, and whether the frequencies have changed during 2001–2009.

### Implications

Although the present study demonstrates a positive trend in adolescents’ eating patterns, our results underline the need for more comprehensive initiatives targeting young people’s food habits. A specific challenge in both Norway and other countries is to increase consumption of vegetables and new approaches might be needed to capture young people’s interest vegetables of such food items. Examples of new and interesting initiatives to communicate with teenagers include the Norwegian Ministry of Health’s use of social media, such as Facebook and Twitter, to spread knowledge and to encourage them to compete in cooking and the creation of healthy recipes. These initiatives are important in order to make meals rich in fruit and vegetables trendy and popular and should be evaluated.

Moreover, there is a need for more deliberate and focused action to close gaps in social inequalities that affect food choices. Norwegian policy action therefore needs to be strengthened and reoriented from what is now primarily “general” coverage to a focus on the specific needs of low SES groups. One example is the government’s suggestion of removing all taxes on fruit, vegetables, which might be particularly relevant to improving food habits among low SES families.

Finally, gender differences in food habits should be taken into account, as school-based interventions aiming to improve health behaviors have been shown to influence boys and girls differently and to favor girls [69,74,75].

Future research should emphasize the need for more systematic evaluation of national policy initiatives, including studies of consumer behavior and the role of structural and educational incentives. Research would also be useful to investigate the effectiveness of the free school fruit scheme, as its continuation is currently under debate by politicians. Moreover, future research should evaluate which factors contribute to social inequalities in food habits, as a basis for the future differentiation of interventions among different target groups.

## Conclusion

The present study show that although the majority of Norwegian adolescents are far from meeting the recommendation of including fruit and vegetables in their everyday diet, the trends are moving in a more health-promoting direction, particularly in the period 2001–2005, in which the survey data registered quite an extensive improvement in fruit and vegetable consumption as well as reduces intake of sweets and soft drinks. Improved food habits were found across SES groups and the socioeconomic differences remained unchanged. This is an encouraging outcome, as improvements in general population health are typically accompanied by increasing inequalities. However, we underscore the need for further initiatives that aim to improve young people’s food habits and reduce socio-economic disparities in fruit and vegetable consumption. Finally, we emphasize the need for future studies of trends in consumption habits to evaluate how young people’s food habits continue to develop.

## Abbreviations

FAS: Family affluence scale
HBSC: Health Behaviour in School-Aged Children
OR: Odds ratio
CI: Confidence interval
SES: Socio-economic status
WHO: World Health Organization
